# Disrupted Spontaneous Neural Activity in Patients With Thyroid-Associated Ophthalmopathy: A Resting-State fMRI Study Using Amplitude of Low-Frequency Fluctuation

**DOI:** 10.3389/fnhum.2021.676967

**Published:** 2021-06-11

**Authors:** Wen Chen, Qian Wu, Lu Chen, Jiang Zhou, Huan-Huan Chen, Xiao-Quan Xu, Hao Hu, Fei-Yun Wu

**Affiliations:** ^1^Department of Radiology, The First Affiliated Hospital of Nanjing Medical University, Nanjing, China; ^2^Department of Endocrinology, The First Affiliated Hospital of Nanjing Medical University, Nanjing, China

**Keywords:** thyroid-associated ophthalmopathy, resting-state functional magnetic resonance imaging, amplitude of low-frequency fluctuation, precuneus, occipital lobe

## Abstract

**Purpose:**

The purpose of the study was to investigate the brain functional alteration in patients with thyroid-associated ophthalmopathy (TAO) by evaluating the spontaneous neural activity changes using resting-state functional magnetic resonance imaging (rs-fMRI) with the amplitude of low-frequency fluctuation (ALFF) method.

**Materials and Methods:**

The rs-fMRI data of 30 TAO patients (15 active and 15 inactive) and 15 healthy controls (HCs) were included for analyses. The ALFF values were calculated and compared among groups. Correlations between ALFF values and clinical metrics were assessed.

**Results:**

Compared with HCs, active TAOs showed significantly decreased ALFF values in the left middle occipital gyrus, superior occipital gyrus, and cuneus. Compared with inactive TAOs, active TAOs showed significantly increased ALFF values in the bilateral precuneus. Additionally, inactive TAOs showed significantly decreased ALFF values in the left middle occipital gyrus, superior occipital gyrus, cuneus, and bilateral precuneus than HCs. The ALFF value in the right precuneus of TAOs was positively correlated with clinical activity score (*r* = 0.583, *P* < 0.001) and Mini-Mental State Examination (MMSE) score (*r* = 0.377, *P* = 0.040), and negatively correlated with disease duration (*r* = −0.382, *P* = 0.037). Moreover, the ALFF value in the left middle occipital gyrus of TAOs was positively correlated with visual acuity (*r* = 0.441, *P* = 0.015).

**Conclusion:**

TAO patients had altered spontaneous brain activities in the left occipital lobe and bilateral precuneus. The neuropsychological aspect of the disease should be noticed during clinical diagnosis and treatment.

## Introduction

Thyroid-associated ophthalmopathy (TAO) is the most common autoimmune inflammatory orbital disease (Sahli and Gunduz, 2017). Although the pathogenesis has not been completely understood, thyrotropin receptors are thought to be the primary target of autoimmune reactions (Hiromatsu et al., 2014). Traditionally, the clinical course of the disease is divided into an acute active phase and a subsequent chronic inactive phase (Drui et al., 2018). During the acute active phase, pathological inflammatory changes, including lymphocyte infiltration, edema, and fibroblast proliferation in orbital tissues, can be present (Hiromatsu et al., 2014), while the chronic inactive phase is characterized by interstitial fibrosis with collagen deposition and fat infiltration (Gould et al., 2012). Due to the acute inflammatory changes in orbital tissues, TAO patients in the active phase usually show good response to anti-inflammatory treatment; however, rehabilitative surgery is suggested for patients in inactive phase because of the steroid resistance to the interstitial fibrosis (Gould et al., 2012).

Clinically, TAO patients usually complain about ophthalmic manifestations as upper eyelid retraction, periorbital edema, exophthalmos, and diplopia (Bahn, 2010; Gould et al., 2012; Hiromatsu et al., 2014; Sahli and Gunduz, 2017). Besides that, TAO patients have also been observed to constantly suffer from series of emotional and psychological abnormalities, including depression, emotional lability, memory deficits, and personality irregularities (Farid et al., 2005; Coulter et al., 2007; Silkiss and Wade, 2016). It was reported that TAO not only restricted patients’ daily activities but also led to dysfunctions in social roles and impaired self-confidence associated with altered appearance (Zeng et al., 2019). The reality for these patients is that their eyes are stared at by strangers (Roos and Murthy, 2019), their identities are changed (Estcourt et al., 2008), and their social functions are disabled (Coulter et al., 2007). Both social isolation and constant ocular disturbances impact the mental state of TAO patients (Kahaly et al., 2002). Evidence has shown that TAO patients underwent higher levels of anxiety and depression than people with other chronic diseases or facial disfigurements (Wickwar et al., 2015). A cohort study even demonstrated a significantly higher risk of suicide (Ferlov-Schwensen et al., 2017). These signs and symptoms have indicated that the patients are not only physically ill but also related to neuropsychic distress. Thus, we believe that TAO may produce neuropathological changes that are responsible for patients’ neuropsychic dysfunctions.

To date, however, a few studies have concentrated on this issue by neuroimaging approaches. A previous structural magnetization-prepared rapid gradient-echo (MPRAGE)-based research by Silkiss et al. (Silkiss and Wade, 2016) demonstrated significant thinning of the gray matter sheet in vision- and cognition-related brain regions in TAO patients. Additionally, a voxel-based morphometry and diffusion tensor imaging study (Wu et al., 2020) also showed that TAO patients had aberrant structural abnormalities in brain areas corresponding to visual and cognitive deficits. Nonetheless, these studies were only focused on structural brain changes, and functional investigation was scarce in this field.

Resting-state functional magnetic resonance imaging (rs-fMRI), a useful technique for exploring intrinsic neural activity via blood oxygen level-dependent signal (Biswal, 2012), has been increasingly utilized in detecting brain functional abnormalities and understanding the neural mechanism of various central nervous system disorders (Leon et al., 2014; Prodoehl et al., 2014; Koch et al., 2016; Takamura and Hanakawa, 2017; Gong et al., 2019; Ma et al., 2019). In recent years, several amplitude methods have been proposed to characterize the local properties of the rs-fMRI signal (Zang et al., 2007; Zou et al., 2008; Jia et al., 2020), and different metrics have shown their own specific characteristics (Zhao et al., 2018; Yu et al., 2019). Among these indices, ALFF provides direct characterization to the spontaneous brain activity at each voxel (Zang et al., 2007; Lv et al., 2019), and has been regarded as one of the most reliable and reproducible rs-fMRI parameters (Liu et al., 2016; Zhao et al., 2018; Zhi et al., 2019). It is obtained by calculating the square root of the power spectrum at each frequency and then averaging the square root across a certain frequency band (Zang et al., 2007). Given the previous findings on clinical psychological manifestations and neuroimaging-based structural changes, we hypothesized that TAO patients would also have spontaneous brain functional changes that might be detected by rs-fMRI with the ALFF method. To our knowledge, the intrinsic resting-state activity changes of TAO have not been investigated using ALFF till now.

Therefore, our study aimed to investigate the brain functional abnormalities in TAO patients by evaluating the spontaneous neural activity changes using rs-fMRI with the ALFF method.

## Materials and Methods

### Subjects

A total of 32 patients with TAO (16 active TAOs and 16 inactive TAOs) were recruited consecutively from the department of endocrinology in our hospital and well matched on sex, age, education level, and handedness. TAO duration was determined from onset of clinical manifestations such as upper eyelid retraction, lid lag, swelling, redness, and proptosis, to date. TAO activity was assessed according to the modified seven-point formulation of Mourits’ clinical activity score (CAS) (Bartalena et al., 2016). Patients with CAS of ≥ 3 were enrolled into the active TAO group; otherwise, they were enrolled into the inactive group. Visual acuity was also performed for each patient. Numerical values of the worse eyes for CAS and visual acuity were recorded. Among all the patients, one active TAO and one inactive TAO were excluded due to poor spatial normalization during data processing. Finally, 30 TAO patients including 15 active TAOs (11 females and 4 males, mean age 44.87 ± 12.44 years) and 15 inactive TAOs (11 females and 4 males, mean age 44.80 ± 11.97 years) were eligible. Concurrently, 15 well-matched healthy controls (HCs) (11 females and 4 males, mean age 44.73 ± 13.24 years) were included. All subjects were in hematologically euthyroid state (TAO group: ≥ 3 months) when they participated in this study (reference ranges: serum free triiodothyronine, 3.10–6.80 pmol/L; free thyronine, 12.00–22.00 pmol/L; thyroid-stimulating hormone, 0.270–4.200 mIU/L). The following exclusion criteria were applied to all subjects: (1) any evidence of other eye diseases (inflammation, orbital tumors, strabismus, amblyopia, cataracts, and glaucoma, etc.), (2) history of eye surgery, (3) history of neurological or psychiatric illness, (4) contraindications to MRI scan, and (5) alcohol or drug addiction. Comorbid depressive and (or) anxious symptoms were not considered as exclusion criteria if TAO was the primary clinical diagnosis. This study was approved by our institutional review board. All subjects volunteered to participate in the study and signed the informed consent.

### Questionnaire Assessments

Life quality and neuropsychological assessments were conducted within 2 h before MRI scan. The English version of Graves’ orbitopathy-specific quality of life (QoL) questionnaire was obtained from the EUGOGO website and translated for TAO patient assessment (Lin et al., 2015). It contained two life quality subscales: visual functioning and appearance. Depression and anxiety symptoms were assessed in all subjects using the 17-item Hamilton Depression Rating Scale (HDRS) and the 14-item Hamilton Anxiety Rating Scale (HARS). Cognitive functions were assessed in all subjects using the Mini-Mental State Examination (MMSE).

### MRI Acquisition

All subjects were examined by using a 3.0-T MR imaging system (MAGNETOM Skyra; Siemens Healthcare, Erlangen, Germany) with a 20-channel head coil. Head motion and scanning noise were reduced by using foam padding and earplugs. The subjects were instructed to lie still in the supine position, close their eyes, relax, and stay awake. High-resolution sagittal structural T1-weighted images were acquired using MPRAGE sequence with the following parameters: repetition time (TR) = 1,900 ms, echo time (TE) = 2.45 ms, flip angle = 9°, field of view (FOV) = 256 × 256 mm^2^, matrix = 256 × 256, thickness = 1.0 mm, number of slices = 176, and voxel size = 1 mm × 1 mm × 1 mm. Functional images were then collected axially by an echo planar imaging sequence with the following parameters: TR = 2,000 ms, TE = 30 ms, flip angle = 90°, FOV = 240 × 240 mm^2^, matrix = 64 × 64, thickness = 4.0 mm, number of slices = 35, and voxel size = 3.75 mm × 3.75 mm × 4 mm. The total scanning duration was 12 min and 26 s.

### Data Preprocessing

All the rs-fMRI data were preprocessed by using RESTplus V1.21^1^ (Jia et al., 2019) based on SPM12^2^ (Ashburner, 2012). The first 10 functional volumes were discarded due to the magnetization equilibration effects and the participants’ adaptation to the scanning environment. Slice timing and realignment for head motion correction were performed. The images were then normalized to the Montreal Neurological Institute template (resampling voxel size = 3 mm × 3 mm × 3 mm) by using T1 image unified segmentation and smoothed with a 6-mm full-width at half-maximum Gaussian kernel. Detrending was applied to remove linear trends. Finally, the nuisance covariates, including the six head motion parameters as well as average signals from cerebrospinal fluid and white matter, were removed by linear regression. If the maximum value of the head translation (rotation) movement was over 2.0 mm (2.0°), the whole dataset of this participant would be discarded. In our study, all the subjects were preserved after head motion correction. To further reduce the influence of head motion, we calculated the mean framewise displacement (FD) based on Jenkinson’s formula (Jenkinson et al., 2002) and included it as a covariate in the following group-level analyses.

### ALFF Calculation

Amplitude of low-frequency fluctuation analysis was also performed by using RESTplus V1.21. The time courses were first transformed into a frequency domain by using the fast Fourier transform algorithm. The average square root of the power spectrum across 0.01–0.08 Hz was calculated as the ALFF value. For standardization purpose, the ALFF values of each voxel were further divided by the global mean of ALFF values (Zang et al., 2007).

### Statistical Analyses

Demographic and clinical data were analyzed using the SPSS software (SPSS 22.0, Inc., Chicago, IL, United States). For continuous variables, one-way analysis of variance (ANOVA) tests (evaluating data with normal distribution) and Kruskal–Wallis tests (evaluating data not normally distributed) were applied to compare the differences among active TAO, inactive TAO and HC groups, while independent-sample *t*-tests (evaluating data with normal distribution) and Mann–Whitney *U*-tests (evaluating data not normally distributed) were applied for differences between any two groups. For categorical variables, Chi-square tests were used. The statistically significant threshold was set at *P* < 0.05.

For the ALFF values, statistical analyses were performed using SPM12. One-way analysis of covariance (ANCOVA) was conducted to compare the group differences of the ALFF values among three groups within a whole-brain mask with age, sex, and mean FD as covariates. Brain areas with significant differences were then extracted as a mask for *post-hoc* analyses. To compare the differences of the ALFF values between any two groups, two-sample *t*-tests were performed within this mask with age, sex, and mean FD as covariates. Statistical significance for both ANCOVA and two-sample *t*-tests was based on a familywise error (FWE) correction for multiple comparisons at the cluster level (P_FWE_ < 0.05) with a cluster-defining threshold of *P* < 0.001, in line with the current reporting guideline (Eklund et al., 2016). The surviving brain regions were visualized using BrainNet Viewer^3^ (Xia et al., 2013) and DPABI^4^ (Yan et al., 2016).

With the peak voxels of significant brain regions in one-way ANCOVA as spherical centers, spherical regions of interest (ROIs) (radius = 6 mm) were constructed, and the ALFF values within these ROIs were extracted for each patient. Spearman’s and Pearson’s correlation analyses were performed to evaluate the relationships between ALFF values and clinical parameters in TAO group (statistical significance was set at uncorrected *P* < 0.05).

### Validation Analyses

The following auxiliary analyses were conducted to evaluate the reproducibility of our findings. First, to examine whether regressing covariates (age, sex, and mean FD) had influence on the results, we performed the statistical analyses without including covariates. Second, considering the relatively small sample size, we applied a “leave-one-out” approach to determine the stability of the identified brain regions. Specifically, in each iteration, one subject was left out, and the remaining subjects were used to repeat the statistical analyses. The significant regions were then saved as a mask, and finally all the masks were added up to see the areas surviving in all iterations.

In addition, given that percent amplitude of fluctuation (PerAF) is a recently proposed amplitude metric and has been proven to show better test–retest reliability (Zhao et al., 2018; Jia et al., 2020), we also computed PerAF for each subject and performed the abovementioned statistical analyses on PerAF with age, sex, and mean FD as covariates. Detailed description of PerAF calculation can be found in previous literature (Zhao et al., 2018).

## Results

### Demographic and Clinical Characteristics

Table 1 lists the demographic and clinical characteristics of all participants. No significant difference was found in age (*P* = 0.964), sex (*P* > 0.999), or educational level (*P* = 0.944) across the three groups. The mean CAS was 3.53 ± 0.74 in active TAOs and 1.53 ± 0.64 in inactive ones (*P* < 0.001). Active TAOs showed significantly shorter disease duration than the inactive group (*P* = 0.023). There was no significant difference in the ratio of antithyroid medication history (*P* = 0.483), the QoL scores for visual functioning (*P* = 0.267), or appearance (*P* = 0.744) between the active and inactive TAO groups. Significant differences were found in the visual acuity (*P* = 0.002) and total scores of HDRS (*P* < 0.001), HARS (*P* < 0.001), and MMSE (*P* = 0.002) among the three groups. Between-group comparisons found that active and inactive TAOs had higher total scores of HDRS and HARS, as well as lower total scores of MMSE and visual acuity than HCs (all *P* < 0.05), while the differences between the two TAO groups were not significant (all *P* > 0.05).

**TABLE 1:** Demographic and clinical characteristics of the active TAO group, inactive TAO group, and HCs.

**Items**	**Active TAOs (*n* = 15)**	**Inactive TAOs (*n* = 15)**	**HCs (*n* = 15)**	**P value**
Age (years)	44.87 ± 12.44	44.80 ± 11.97	44.73 ± 13.24	0.964^a#^
Sex (female/male)	11/4	11/4	11/4	> 0.999^b^
Education (years)	12.80 ± 3.90	12.87 ± 5.32	13.00 ± 3.93	0.944^a#^
Handedness (right/left)	15/0	15/0	15/0	> 0.999^b^
Disease duration (months)	9.13 ± 7.77	25.20 ± 24.31	–	0.023^c^
Antithyroid medication	13 (86.7%)	15 (100.0%)	–	0.483^b^
CAS	3.53 ± 0.74	1.53 ± 0.64	–	< 0.001^c^
Visual acuity	0.77 ± 0.15	0.81 ± 0.16	0.98 ± 0.16	0.002^d*^
**QoL scores**				
Visual functioning	59.40 ± 32.69	70.95 ± 29.51	–	0.267^c^
Appearance	69.12 ± 26.70	71.25 ± 18.11	–	0.744^c^
Total score of HDRS	15.00 ± 9.67	16.07 ± 9.13	2.00 ± 1.65	< 0.001^a*^
Total score of HARS	14.93 ± 9.08	16.93 ± 6.68	2.40 ± 1.35	< 0.001^a*^
Total score of MMSE	28.47 ± 1.92	27.80 ± 2.34	29.87 ± 0.35	0.002^a*^

Data were presented as mean ± standard deviation unless otherwise indicated. CAS, clinical activity score; QoL, quality of life; HDRS, Hamilton Depression Rating Scale; HARS, Hamilton Anxiety Rating Scale; MMSE, Mini-Mental State Examination; TAO, thyroid-associated ophthalmopathy; HCs, healthy controls; n, number of subjects. ^*a*^*P* value with Kruskal–Wallis test. ^*b*^*P* value with Chi-square test. ^*c*^*P* value with Mann–Whitney test. ^*d*^*P* value with one-way ANOVA test. In addition, ^#^ indicates that there was no difference between any two groups, while * indicates that for all *post hoc* tests, active and inactive TAO groups differed significantly from HCs, but the difference between the active TAO group and inactive TAO group were not statistically significant.

### ALFF Analyses

**FIGURE 1:**
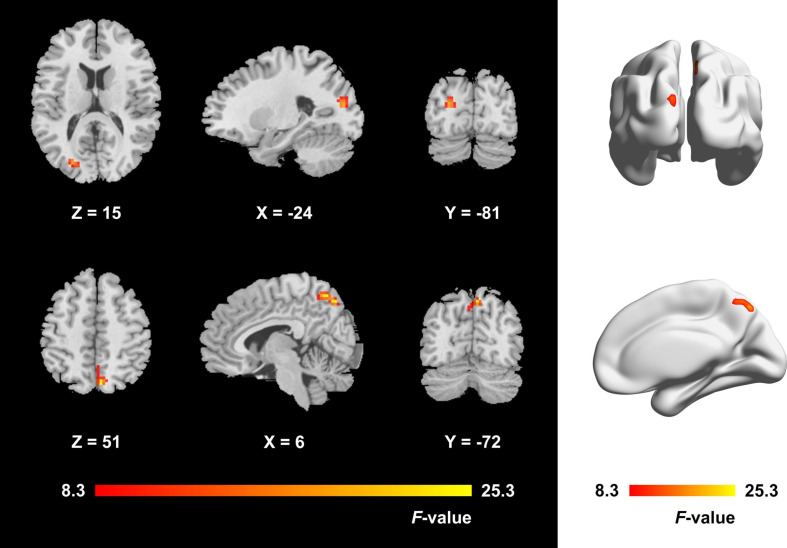
Brain regions with significant ALFF difference among active TAO group, inactive TAO group, and HCs. The differences primarily existed in the left middle occipital gyrus, superior occipital gyrus, cuneus, and bilateral precuneus (voxel *P* < 0.001, cluster *P* < 0.05, cluster-level FWE corrected, cluster size ≥ 67 voxels). The color bar indicates the F value from analysis of covariance among the three groups. ALFF, amplitude of low frequency fluctuation; TAO, thyroid-associated ophthalmopathy; HCs, healthy controls; FWE, familywise error.

**FIGURE 2:**
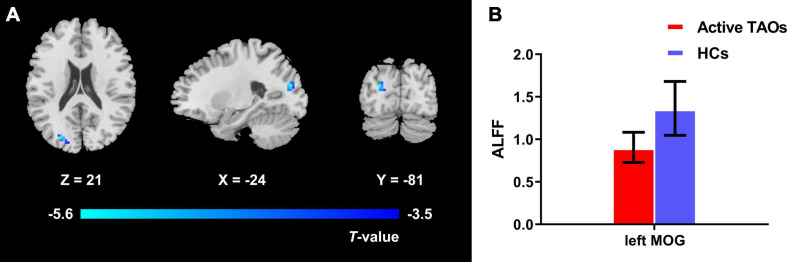
**(A)** Brain regions with significant ALFF difference between active TAO group and HCs. Compared with HCs, active TAO group showed significantly decreased ALFF values in the left middle occipital gyrus, superior occipital gyrus, and cuneus (voxel *P* < 0.001, cluster *P* < 0.05, cluster-level FWE corrected, cluster size ≥ 48 voxels). Cold color denotes relatively lower ALFF values in the active TAO group, and the color bar indicates the T value from the two-sample *t*-test between the active TAO group and HCs. **(B)** The bar plot demonstrates the ALFF difference of significant brain regions in active TAO group and HCs. The ALFF values used were the mean ALFF values extracted by using an ROI defined as a sphere centered at the peak voxel with a 6-mm radius. ALFF, amplitude of low frequency fluctuation; TAO, thyroid-associated ophthalmopathy; HCs, healthy controls; FWE, familywise error; ROI, region of interest; MOG, middle occipital gyrus.

**FIGURE 3:**
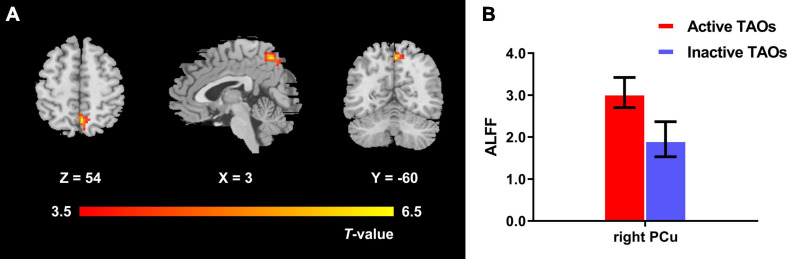
**(A)** Brain regions with significant ALFF difference between active and inactive TAO groups. Compared with the inactive TAO group, the active TAO group showed significantly increased ALFF values in the bilateral precuneus (voxel *P* < 0.001, cluster *P* < 0.05, cluster-level FWE corrected, cluster size ≥ 64 voxels). Warm color denotes relatively higher ALFF values in active TAO group, and the color bar indicates the T value from the two-sample *t*-test between the active and inactive TAO group. **(B)** The bar plot demonstrates the ALFF difference of significant brain regions in the active and inactive TAO groups. The ALFF values used were the mean ALFF values extracted by using an ROI defined as a sphere centered at the peak voxel with a 6-mm radius. ALFF, amplitude of low frequency fluctuation; TAO, thyroid-associated ophthalmopathy; FWE, familywise error; ROI, region of interest; PCu, precuneus.

**FIGURE 4:**
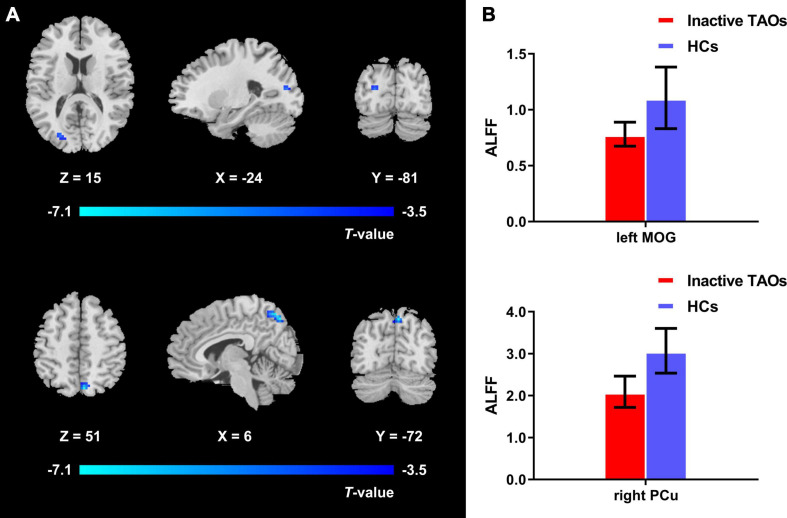
**(A)** Brain regions with significant ALFF difference between the inactive TAO group and HCs. Compared with HCs, the inactive TAO group showed significantly decreased ALFF values in the left middle occipital gyrus, superior occipital gyrus, cuneus, and bilateral precuneus (voxel *P* < 0.001, cluster *P* < 0.05, cluster-level FWE corrected, cluster size ≥ 34 voxels). Cold color denotes relatively lower ALFF values in the inactive TAO group, and the color bar indicates the *T* value from the two-sample *t*-test between the inactive TAO group and HCs. **(B)** The bar plot demonstrates the ALFF difference of the significant brain regions in the inactive TAO group and HCs. The ALFF values used were the mean ALFF values extracted by using an ROI defined as a sphere centered at the peak voxel with a 6-mm radius. ALFF, amplitude of low frequency fluctuation; TAO, thyroid-associated ophthalmopathy; HCs, healthy controls; FWE, familywise error; ROI, region of interest; MOG, middle occipital gyrus; PCu, precuneus.

The one-way ANCOVA results showed that significant ALFF differences among the three groups primarily existed in the left middle occipital gyrus, superior occipital gyrus, cuneus, and bilateral precuneus (voxel *P* < 0.001, cluster *P* < 0.05, cluster-level FWE corrected, cluster size ≥ 67 voxels) (Figure 1). For pairwise comparisons, the active TAO group showed significantly decreased ALFF values in the left middle occipital gyrus, superior occipital gyrus, and cuneus (voxel *P* < 0.001, cluster *P* < 0.05, cluster-level FWE corrected, cluster size ≥ 48 voxels) than HCs (Figure 2). Compared with inactive TAOs, active TAOs showed significantly increased ALFF values in the bilateral precuneus (voxel *P* < 0.001, cluster *P* < 0.05, cluster-level FWE corrected, cluster size ≥ 64 voxels) (Figure 3). As to those between inactive TAO and HC groups, inactive TAOs showed significantly decreased ALFF values in the left middle occipital gyrus, superior occipital gyrus, cuneus, and bilateral precuneus (voxel *P* < 0.001, cluster *P* < 0.05, cluster-level FWE corrected, cluster size ≥ 34 voxels) than HCs (Figure 4). The detailed information for all brain regions with significant ALFF values among the three groups is shown in Table 2.

**TABLE 2:** Brain areas with significantly different ALFF values between groups (voxel *P* < 0.001, cluster *P* < 0.05, cluster-level FWE corrected).

**Brain regions/conditions**	**BA**	**MNI coordinates**			**Cluster size (number of voxels)**	***t*-value**
		**X**	**Y**	**Z**		
**Active TAOs < HCs**						
Left middle occipital gyrus/Left superior occipital gyrus/Left cuneus	18/19	−24	−81	21	48	−5.612
**Active TAOs > Inactive TAOs**						
Bilateral precuneus	7	3	−60	54	64	6.505
**Inactive TAOs < HCs**						
Left middle occipital gyrus/Left superior occipital gyrus/Left cuneus	18/19	−24	−81	15	34	−4.798
Bilateral precuneus	7	6	−72	51	56	−7.119

*BA, Brodmann’s areas; MNI, Montreal Neurologic Institute; TAO, thyroid-associated ophthalmopathy; HCs, healthy controls; ALFF, amplitude of low frequency fluctuation; FWE, familywise error.*

### Correlations Between Amplitude of Low-Frequency Fluctuation and Clinical Measures

**FIGURE 5:**
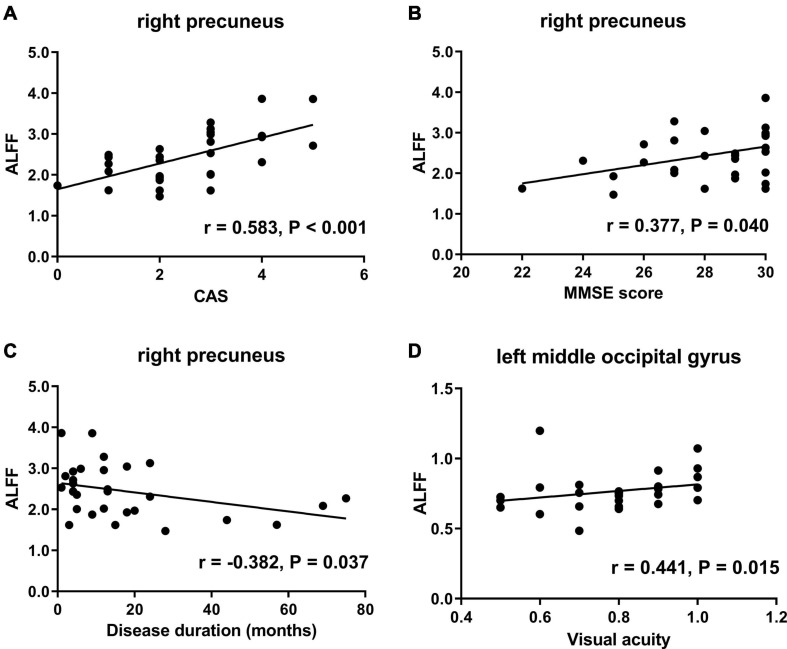
Scatter diagrams show the significant correlations between clinical and neuropsychological assessment results and the ALFF values in TAO patients. **(A)** ALFF in the right precuneus was positively correlated with CAS (*r* = 0.583, *P* < 0.001). **(B)** ALFF in the right precuneus was positively correlated with MMSE score (*r* = 0.377, *P* = 0.040). **(C)** ALFF in the right precuneus was negatively correlated with disease duration (*r* = −0.382, *P* = 0.037). **(D)** ALFF in the left middle occipital gyrus was positively correlated with visual acuity (*r* = 0.441, *P* = 0.015). ALFF, amplitude of low frequency fluctuation; TAO, thyroid-associated ophthalmopathy; CAS, clinical activity score; MMSE, Mini-Mental State Examination.

The correlation analyses revealed that the ALFF values in the right precuneus of TAO patients were positively correlated with CAS (*r* = 0.583, *P* < 0.001) and MMSE score (*r* = 0.377, *P* = 0.040) and negatively correlated with disease duration (*r* = −0.382, *P* = 0.037) (Figures 5A–C). Moreover, the ALFF values in the left middle occipital gyrus of TAO patients were positively correlated with visual acuity (*r* = 0.441, *P* = 0.015) (Figure 5D). No significant correlation was found between ALFF values with any other clinical measures, including QoL, HDRS, and HARS scores.

### Validation Analyses

To evaluate the possible influence of regressing covariates (age, sex, and mean FD) on the results, we repeated the statistical analyses without including covariates. The brain regions with significant ALFF difference without including covariates were consistent with those in the main findings (Supplementary Table 1 and Supplementary Figure 1), which indicated that the observed ALFF alterations were independent of the included covariates.

To verify the stability of our findings, we conducted additional analyses using the “leave-one-out” approach. The brain regions showing significant difference were largely preserved, although the areas surviving in all iterations were smaller than those in the main results (Supplementary Table 2 and Supplementary Figure 2). This finding suggested that the identified brain regions were stable in the present dataset.

We also computed and analyzed the PerAF metric using our dataset. However, no significant difference was found among the three groups after multiple comparison correction (voxel *P* < 0.001, cluster *P* < 0.05, cluster-level FWE corrected).

## Discussion

To our knowledge, the present study is the first to investigate the brain activity alteration in TAO patients with different stages. Our study initially used the ALFF method based on rs-fMRI to explore the brain functional changes in TAO patients and demonstrated that TAOs had altered spontaneous brain activities in the left occipital lobe and bilateral precuneus. In the TAO group, ALFF in the left middle occipital gyrus was correlated with visual acuity, while ALFF in the right precuneus was correlated with CAS, MMSE scores, and disease duration. These findings may improve our understanding on the neural mechanism of the disease.

In this study, the active TAO group demonstrated decreased ALFF values in the left middle occipital gyrus, superior occipital gyrus, and cuneus compared with HCs. The occipital lobe is a key brain region for visual processing, mainly involved in visual formation and functional activities of visual perception (Yu et al., 2017). Therefore, disruption of this region might reflect alteration in visual function. Disrupted brain activity of the occipital lobe has been observed in neuroimaging studies concerning amblyopia (Dai et al., 2019), blindness (Huang et al., 2019), and glaucoma (Chen et al., 2017; Jiang et al., 2019). Similar to these findings, decreased ALFF in the left occipital lobe in active TAOs may provide a neural basis for disrupted visual processing in this disease. Our results also indicated that the brain areas with reduced ALFF in TAO were mainly located in Brodmann areas (BA) 18 and 19. BA 18 and 19 contain visual association areas (V2 and V3), which are responsible for early-stage perceptive analysis of visual information (Lu et al., 2019). Given that active-phase TAO manifests as chemosis, swollen caruncle, proptosis, and eyelid swelling, with diplopia as one of its most common and debilitating symptoms, it would be a reasonable finding that active TAOs had significant brain activity changes in the occipital visual cortex. Combining the significantly positive correlation with visual acuity, we could further deduce that brain activity disturbance in the occipital brain region might progress along with the reduced visual function. In addition, research by Schraa-Tam et al. (2009) showed that the cuneus was involved in eye movement reflex, which functioned to stabilize the image of the retina. Thus, dysfunction of this region could affect eye movement regulation. We consider that the aberrant ALFF in the left cuneus may also indicate the aberrant neural activity change related to eye movement restriction in TAO patients.

Another important finding of our study is the decreased ALFF in the bilateral precuneus of inactive TAOs compared with active ones. The precuneus, located in the medial wall of BA7, is one of the brain regions with the highest metabolic rate (Zhu et al., 2012) and forms an important part of the default mode network (Buckner et al., 2008; Fransson and Marrelec, 2008; Utevsky et al., 2014). It plays a critical role in various complex functions, such as recollection and memory, self-reflection, consciousness, and linking new information with experience (Lou et al., 2004; Lundstrom et al., 2005; Cavanna and Trimble, 2006). Therefore, the precuneus involves in a wide variety of high-level cognitive processes. Significant brain activity changes in the precuneus have been reported in mild cognitive impairment (Pan et al., 2017; Luo et al., 2018) and Alzheimer’s disease (Bailly et al., 2015). In line with these studies, our results of decreased ALFF in the right precuneus in inactive TAOs and its positive correlation with MMSE scores (range, 22–30) might provide potential evidence of cognitive dysfunctions. In addition, decreased ALFF in the right precuneus was positively correlated with CAS and negatively correlated with disease duration. Our further analysis showed that CAS was significantly correlated with disease duration (*r* = −0.474, *P* = 0.008). Considering the clinical basis of the disease, we hypothesize that the cognitive disturbance might progress along with the disease process. With the extension of disease duration, the disease activity regressed, while the cognitive decline gradually appeared.

As to the comparison between inactive TAO and HC groups, decreased ALFF values in the left occipital lobe and bilateral precuneus were observed concurrently. This finding, integrated with those of the active TAOs, indicated that neural activity changes of TAO might be a progressive process, in which early active TAO would mainly show disruption in visual cortex, while the chronic inactive phase would further demonstrate brain functional abnormality concerning early cognitive impairment. The underlying mechanism of cognitive dysfunctions in TAO remains unclear till now. Previous studies showed that impaired self-confidence and emotional problems may impact cognitive functions (Brosch et al., 2013; Duclos et al., 2018). Given that TAO patients could suffer from emotional problems, dysfunction in social roles, and impaired self-confidence because of altered appearance (Farid et al., 2005; Coulter et al., 2007; Silkiss and Wade, 2016; Zeng et al., 2019), the cognitive impairment might be secondary to these psychological abnormalities. In addition, dysfunction in thyroid hormones was also documented to lead to cognitive deficit (Bauer et al., 2008; Ritchie and Yeap, 2015). Although all the TAO patients were in a hematologically euthyroid state when they participated in our research, prior abnormal thyroid hormone levels could have already impacted their brain functions. The precise neuromechanism of cognitive dysfunctions in TAO remains to be elucidated in future research.

Nonetheless, our study, based on rs-fMRI using the ALFF method, is an extension to previous structural imaging-based analyses. The identified significant brain regions were highly consistent with those in the structural investigations by Silkiss et al. (Silkiss and Wade, 2016) and Wu et al. (2020). Therefore, we believe that the aberrant functional activities of occipital lobe and precuneus might be the prominent neuroimaging markers of TAO. Our results further suggest that TAO could be a somatopsychic disease, and its psychological aspect should also be noted during clinical diagnosis and treatment.

In this study, we did not find any significant correlations between ALFF in the left occipital lobe or the right precuneus with QoL scores. Some previous studies reported low correlation between QoL scores and clinical activity of TAO, and explained that QoL was a subjective and rough measure of patients’ experiences rather than objective and detailed clinical measures (Park et al., 2004; Yeatts, 2005). We speculate that our non-significant results could also be attributed to a similar reason.

Percent amplitude of fluctuation is a recently proposed amplitude metric with better test–retest reliability (Zhao et al., 2018; Jia et al., 2020). Studies have shown that PerAF is more sensitive than ALFF (Zhao et al., 2018; Yu et al., 2019; Jia et al., 2020); however, in contrast to the observed ALFF differences, we did not find significant PerAF alteration based on our present dataset. We speculate that this discrepancy might be associated with the distinct sample sizes and statistical methods of different studies. Future investigations with larger cohort and more comprehensive dataset of diseases are warranted to validate this issue.

The present study has several limitations. First, it was a preliminary study with relatively small sample size. Future studies with more patients could strengthen the statistical power and verify our results. Second, although the TAO patients we enrolled were in short-term euthyroid state, it is impossible to completely circumvent the potential impact of thyroid hormone in brain functional changes. Grouping primarily euthyroid- and hyperthyroid-associated TAO patients might be helpful to verify our findings. Third, in addition to the spontaneous neural activity over the whole brain, functional connectivity measurements should also be included to obtain more comprehensive findings regarding the correlations between different brain regions. Fourth, the MMSE used for cognitive assessment in this study may not fully reflect the different aspects of cognitive functions. Various cognitive domain scales should be employed to detect early dysfunctions in future investigations. Last, this is a cross-sectional study, and future follow-up research with larger cohorts of patients is needed to expand our understanding in the neural activity changes of TAO.

## Conclusion

Our study based on rs-fMRI with ALFF indicated that TAO patients had altered spontaneous brain activities in the left occipital lobe and bilateral precuneus. The active phase was associated with changes in the occipital visual cortex, while the inactive phase further impacted the precuneus concerning potentially early cognitive impairment. These findings provided novel insights into brain function abnormalities in TAO patients and indicated that the neuropsychological aspect of the disease should also be noticed in clinical practice.

## Data Availability Statement

The raw data supporting the conclusions of this article will be made available by the authors, without undue reservation.

## Ethics Statement

The studies involving human participants were reviewed and approved by the Ethics Committee of the First Affiliated Hospital of Nanjing Medical University. The patients/participants provided their written informed consent to participate in this study.

## Author Contributions

HH, X-QX, and F-YW conceptualized and designed the study. WC, QW, LC, and JZ performed the MR scan. WC performed the MR data analyses and wrote the first draft. H-HC contributed to the diagnosis and clinical data collection. HH provided critical revisions of the draft. All authors approved the manuscript for submission.

## Conflict of Interest

The authors declare that the research was conducted in the absence of any commercial or financial relationships that could be construed as a potential conflict of interest.

## Footnotes

1 http://www.restfmri.net/forum/RESTplusV1.2

2 http://www.fil.ion.ucl.ac.uk/spm/software/spm12

3 http://www.nitrc.org/projects/bnv

4 http://www.rfmri.org/dpabi
