# Supplementary material for: Disrupted Spontaneous Neural Activity in Patients With Thyroid-Associated Ophthalmopathy: A Resting-State fMRI Study Using Amplitude of Low-Frequency Fluctuation
Source: Front Hum Neurosci. 2021 Jun 11;15:676967. doi: 10.3389/fnhum.2021.676967 (PMC8226248; doi:10.3389/fnhum.2021.676967)
Supplement: Supplementary file 1 [file Data_Sheet_1.docx]

***Supplementary Material***

# Supplementary Tables

**Table S1.** Brain areas with significantly different ALFF values between groups (without including covariates) (voxel P < 0.001, cluster P < 0.05, cluster-level FWE corrected)

| **Brain regions/**  **conditions** | **BA** | **MNI coordinates** | | | **Cluster size**  **(number of voxels)** | **t-value** |
| --- | --- | --- | --- | --- | --- | --- |
|  |  | X | Y | Z |  |  |
| **Active TAOs < HCs** |  |  |  |  |  |  |
| **Left middle occipital gyrus/**  **Left superior occipital gyrus/**  **Left cuneus** | 18/19 | -24 | -81 | 21 | 68 | -5.862 |
| **Active TAOs > Inactive TAOs** |  |  |  |  |  |  |
| **Bilateral precuneus** | 7 | 3 | -60 | 54 | 87 | 7.512 |
| **Inactive TAOs < HCs** |  |  |  |  |  |  |
| **Left middle occipital gyrus/**  **Left superior occipital gyrus/**  **Left cuneus** | 18/19 | -24 | -81 | 15 | 34 | -4.810 |
| **Bilateral precuneus** | 7 | 6 | -63 | 57 | 56 | -6.354 |

BA, Brodmann’s areas; MNI, Montreal Neurologic Institute; TAO, thyroid-associated ophthalmopathy; HCs, healthy controls; ALFF, amplitude of low frequency fluctuation; FWE, familywise error

**Table S2.** Brain areas surviving in all iterations of the “leave-one-out” validation (voxel P < 0.001, cluster P < 0.05, cluster-level FWE corrected)

| **Brain regions/**  **conditions** | **BA** | **Cluster size**  **(number of voxels)** |
| --- | --- | --- |
| **Active TAOs < HCs** |  |  |
| **Left middle occipital gyrus/**  **Left superior occipital gyrus/**  **Left cuneus** | 18/19 | 32 |
| **Active TAOs > Inactive TAOs** |  |  |
| **Bilateral precuneus** | 7 | 41 |
| **Inactive TAOs < HCs** |  |  |
| **Left middle occipital gyrus** | 19 | 10 |
| **Left superior occipital gyrus/**  **Left cuneus** | 18 | 7 |
| **Right precuneus** | 7 | 41 |

BA, Brodmann’s areas; TAO, thyroid-associated ophthalmopathy; HCs, healthy controls; FWE, familywise error

# Supplementary Figures


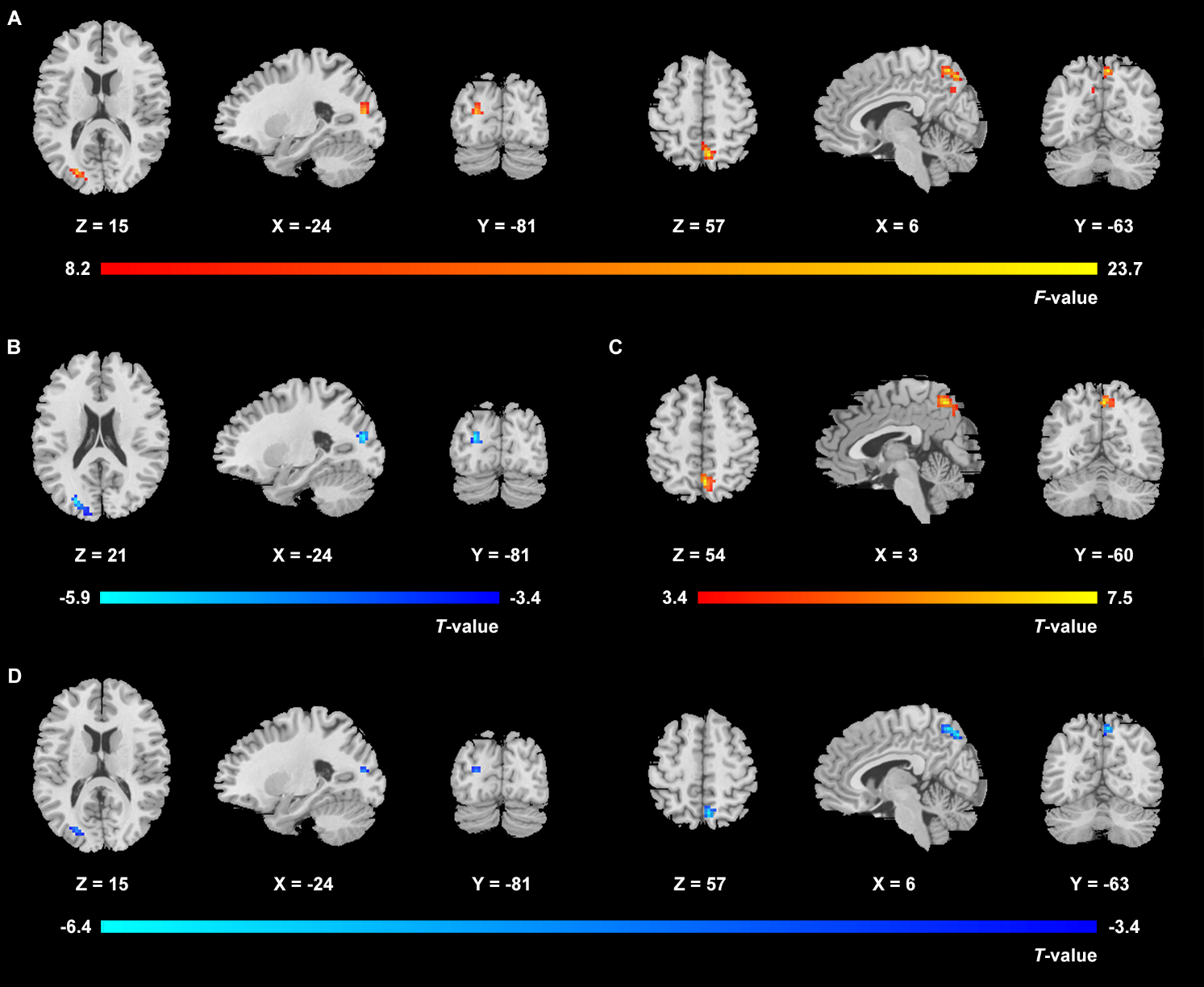


**Figure S1.** Brain regions with significant ALFF difference across active TAO group, inactive TAO group and HCs based on One‑way ANOVA (A) and post-hoc two‑sample t‑tests (B, C and D) (without including covariates). (A) The differences among the three groups primarily existed in the left middle occipital gyrus, superior occipital gyrus, cuneus and bilateral precuneus (voxel P < 0.001, cluster P < 0.05, cluster-level FWE corrected, cluster size ≥ 73 voxels). (B) Compared with HCs, active TAO group showed significantly decreased ALFF values in the left middle occipital gyrus, superior occipital gyrus and cuneus (voxel P < 0.001, cluster P < 0.05, cluster-level FWE corrected, cluster size ≥ 68 voxels). (C) Compared with inactive TAO group, active TAO group showed significantly increased ALFF values in the bilateral precuneus (voxel P < 0.001, cluster P < 0.05, cluster-level FWE corrected, cluster size ≥ 87 voxels). (D) Compared with HCs, inactive TAO group showed significantly decreased ALFF values in the left middle occipital gyrus, superior occipital gyrus, cuneus and bilateral precuneus (voxel P < 0.001, cluster P < 0.05, cluster-level FWE corrected, cluster size ≥ 34 voxels). The color bar indicates the F/T value among/between groups, and the warm and cold colors denote significantly increased and decreased values. ALFF, amplitude of low frequency fluctuation; TAO, thyroid-associated ophthalmopathy; HCs, healthy controls; ANOVA, analysis of variance; FWE, familywise error.


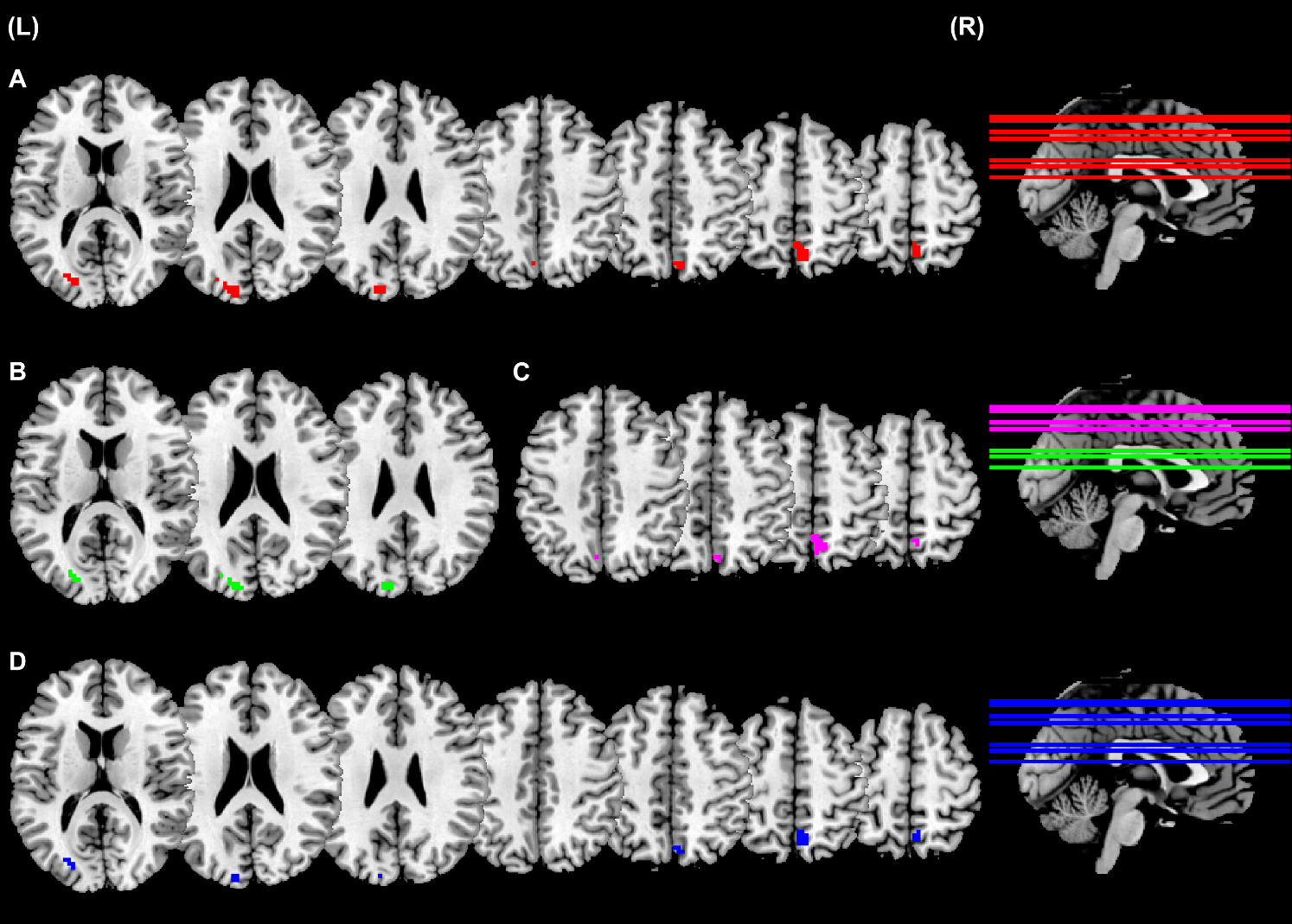


**Figure S2.** Brain regions surviving in all iterations of the “leave-one-out” validation. (A) For one‑way ANCOVA, the regions surviving in all iterations were the left middle occipital gyrus, superior occipital gyrus, cuneus and bilateral precuneus (red color). (B) For post-hoc inter-group comparisons between active TAOs and HCs, the regions surviving in all iterations were the left middle occipital gyrus, superior occipital gyrus and cuneus (green color). (C) For post-hoc inter-group comparisons between active and inactive TAOs, the regions surviving in all iterations were the bilateral precuneus (violet color). (D) For post-hoc inter-group comparisons between inactive TAOs and HCs, the regions surviving in all iterations were the left middle occipital gyrus, superior occipital gyrus, cuneus and right precuneus (blue color). ANCOVA, analysis of covariance; TAO, thyroid-associated ophthalmopathy; HCs, healthy controls; L, left; R, right.
